# Isolation of Flaviviruses and Alphaviruses with Encephalitogenic Potential Diagnosed by Evandro Chagas Institute (Pará, Brazil) in the Period of 1954–2022: Six Decades of Discoveries

**DOI:** 10.3390/v15040935

**Published:** 2023-04-10

**Authors:** Ana Lucia Monteiro Wanzeller, Fabio Silva da Silva, Leonardo Henrique Almeida Hernández, Landerson Junior Leopoldino Barros, Maria Nazaré Oliveira Freitas, Maissa Maia Santos, Ercília de Jesus Gonçalves, Jamilla Augusta Sousa Pantoja, Creuza de Sousa Lima, Maxwell Furtado Lima, Luiz Roberto Oliveira Costa, Liliane Leal das Chagas, Iveraldo Ferreira Silva, Tania Cristina Alves da Silveira da Cunha, Bruna Lais Sena do Nascimento, Helena Baldez Vasconcelos, Elizabeth Salbe Travassos da Rosa, Sueli Guerreiro Rodrigues, Raimunda do Socorro da Silva Azevedo, Lívia Carício Martins, Lívia Medeiros Neves Casseb, Jannifer Oliveira Chiang, Joaquim Pinto Nunes Neto, Ana Cecília Ribeiro Cruz, Valéria Lima Carvalho, Pedro Fernando da Costa Vasconcelos, Eliana Vieira Pinto da Silva

**Affiliations:** Viral Isolation Laboratory, Department of Arbovirology and Hemorrhagic Fevers, Evandro Chagas Institute, Secretariat of Health and Environment Surveillance, Ministry of Health, Ananindeua 67030-000, Brazil

**Keywords:** arbovirus, viral isolation, cell culture, neurotropic, public health

## Abstract

Viruses with encephalitogenic potential can cause neurological conditions of clinical and epidemiological importance, such as *Saint Louis encephalitis virus*, *Venezuelan equine encephalitis virus*, *Eastern equine encephalitis virus*, *Western equine encephalitis virus*, *Dengue virus*, *Zika virus*, *Chikungunya virus*, *Mayaro virus* and *West Nile virus*. The objective of the present study was to determine the number of arboviruses with neuroinvasive potential isolated in Brazil that corresponds to the collection of viral samples belonging to the Department of Arbovirology and Hemorrhagic Fevers, Evandro Chagas Institute (SAARB/IEC) of the Laboratory Network of National Reference for Arbovirus Diagnosis from 1954 to 2022. In the analyzed period, a total of 1,347 arbovirus samples with encephalitogenic potential were isolated from mice; 5,065 human samples were isolated exclusively by cell culture; and 676 viruses were isolated from mosquitoes. The emergence of new arboviruses may be responsible for diseases still unknown to humans, making the Amazon region a hotspot for infectious diseases due to its fauna and flora species characteristics. The detection of circulating arboviruses with the potential to cause neuroinvasive diseases is constant, which justifies the continuation of active epidemiological surveillance work that offers adequate support to the public health system regarding the virological diagnosis of circulating arboviruses in Brazil.

## 1. Introduction

For more than 80 years, the Evandro Chagas Institute (IEC), through a monitoring program for the collection of virological and serological data, has isolated and classified, completely or partially, about 200 different types of arboviruses pathogenic for humans [1,2]. Some viruses discovered by this program still lack a completely defined taxonomic classification.

The term arbovirus is derived from the union of the first syllables of the English expression arthropod-borne (transmitted by arthropods) followed by the suffix virus. According to the concept established by the World Health Organization (WHO) [3], “Arboviruses are viruses maintained in nature through biological transmission between susceptible vertebrate hosts and hematophagous arthropods, or between arthropod hosts by the transovarian and/or venereal route. They are able to reproduce in vertebrate hosts, multiply in arthropod tissues and be passed on to new susceptible vertebrates by arthropod bites after an extrinsic incubation period”.

Arbovirus infections can result in a wide spectrum of clinical syndromes, from mild febrile illness to hemorrhagic fevers and neuroinvasive forms. However, most human arbovirus infections are asymptomatic or oligosymptomatic [4].

The Centers for Disease Control and Prevention (CDC) defines meningitis (aseptic), encephalitis and acute flaccid paralysis as the main neurological manifestations of arbovirus infection, although many cases are not recognized as manifestations caused by these viruses, which contributes to the diagnosis remaining a challenge to be faced [5].

Population growth, disorderly urbanization and increased human traffic and international trade have contributed to the emergence and spatial spread of arboviruses in recent decades [6,7]. Every day, other forms of arbovirus transmission, such as blood transfusion, organ transplantation, sexual or perinatal transmission and laboratory exposure, gain importance [8].

Currently, approximately seven viral families (*Peribunyaviridae*, *Togaviridae*, *Flaviviridae*, *Sedoreoviridae*, *Rhabdoviridae*, *Asfaviridae* and *Phenuiviridae*) harbor arboviruses capable of infecting humans and animals and have become important in public health [9,10,11,12], with emphasis on the *Dengue virus* (DENV), the *Chikungunya virus* (CHIKV), the *Zika virus* (ZIKV), the *West Nile virus* (WNV) and the *Rocio virus* (ROCV) as neuroinvasive viruses of the greatest epidemiological interest in Brazil [4].

**Figure 1:**
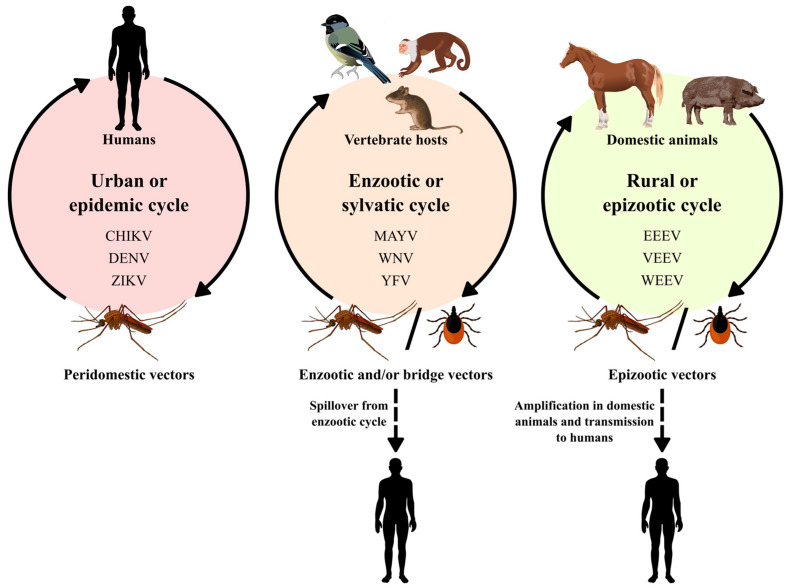
Alphaviruses and flaviviruses transmission cycles.

The *Alphavirus* genus belongs to the *Togaviridae* family, which is composed of 32 viruses, including the *Mayaro virus* (MAYV), the *Eastern equine encephalitis virus* (EEEV), the *Chikungunya virus* (CHIKV) and the *Western equine encephalitis virus* (WEEV), which are important in public health. Additionally, the *Aura virus* (AURAV), the *Una virus* (UNAV), the *Mucambo virus* (MUCV), the *Triniti virus* (TNTV) and the *Pixuna virus* (PIXV) are also included [13]. Alphaviruses are approximately 70 nm in diameter, with an icosahedral capsid enveloped with positive-sense, single-stranded RNA genomes ranging from 10 to 12 kb [14]. These viruses are transmitted by mosquitoes, although other blood-sucking insects, including ticks, lice and bed bugs, have been implicated in transmission [15,16]. Vertebrate hosts include humans, nonhuman primates, equids, birds, amphibians, reptiles, rodents and pigs (Figure 1) [17].

The *Flavivirus* genus belongs to the *Flaviviridae* family and is composed of 53 viral representatives, including the *Japanese encephalitis virus* (JEV), DENV, ZIKV, and WNV, of public health importance. The following viruses are also included: the *Cacipacoré virus* (CACV), the *Ilhéus virus* (ILHV), the *Bussuquara virus* (BUSV) and the *Iguape virus* (IGUV) [13]. Flaviviruses have positive-sense RNA of approximately 9.0–13 kb with an icosahedral symmetrical structure with an approximate diameter of 40 to 60 nm [18].

Flaviviruses are transmitted by arthropod vectors through transovarian or vertical transmission and by cycles between vectors and vertebrate hosts. Ticks also contribute to the maintenance of these viruses via vertical and transstadial routes. Rodents and bats can also be part of the *Flavivirus* transmission cycle, with no known arthropod vectors. Humans are not definitive hosts but accidental hosts when they enter the habitat of these mosquitoes (Figure 1). In certain cases, flaviviruses can be transmitted to humans by blood products, organ transplantation, unpasteurized milk or aerosols. It is worth mentioning that individuals who move to areas endemic with these vectors are at greater risk of acquiring the disease [18].

### 1.1. Alphavirus Genus

The *Togaviridae* family includes the *Alphavirus* genus comprising 32 viral species [19], with a single-stranded RNA genome, spherical and enveloped. The envelope contains spikes of glycoproteins (E1 and E2). The viral particle measures 70 nm in diameter and infects vertebrates and invertebrates. The alphaviruses include mosquito-transmitted diseases such as Eastern, Western, or Venezuelan equine encephalitis, which are characterized by fever, malaise, headache and encephalitis, and diseases caused by the *Chikungunya virus*, the *Ross River virus*, the *Mayaro virus* and the *Sindbis virus*, which produce arthralgias. The syndrome produced by viruses, such as the *Chikungunya virus,* result in intense joint pain, high fevers and a rash, and although infection is self-limited, symptoms may persist for years. In addition to mosquito transmission, the virus can be transmitted vertically as well as with infected blood transfusions [14,17].

#### 1.1.1. Mayaro Virus (MAYV)

MAYV originates from the Americas and was first isolated in 1954 in Trinidad and Tobago, islands in the Caribbean region, and was also isolated in primates and migratory birds in the United States. In Brazil, the first identification took place in 1955 during an outbreak of an acute febrile illness with headache that affected rural workers on a farm located on the left banks of the Guamá River in Belém. After three decades, new outbreaks were confirmed in localities in Pará, such as Benevides (1991), Belterra (1978), Santa Bárbara and Conceição do Araguaia (1981) [20,21,22,23,24].

In a recent epidemiological investigation, MAYV was detected in human populations residing in the municipalities of Parauapebas and Canaã dos Carajás in the Pará state, evidencing the circulation of arboviruses in this area, which reinforces the importance of epidemiological surveillance in areas that are under pressure from environmental changes [25].

The transmission of MAYV occurs through the bite of a female mosquito belonging to the genus *Haemagogus*, particularly the species *Haemagogus janthinomys* [26], which has diurnal and wild habits and lives in treetops, facilitating its contact with the host and consequently its transmission. The global expansion of the two main urban vectors (*Aedes aegypti* and *Aedes albopictus*) and the increasing number of international travelers certainly contribute to the increased risk of MAYV transmission [27].

In the case of viral infection, variations in the habitats and behaviors of viruses have been noted in recent decades, such as the spread of MAYV by infected birds or humans, resulting in a transmission cycle that may involve humans as a reservoir [28].

Considering the lack of licensed vaccines and proven therapeutics, prevention focuses mainly on domestic mosquito control to contain the spread of MAYV [29].

#### 1.1.2. Eastern Equine Encephalitis Virus (EEEV)

This encephalitis manifests itself with severe neurological conditions in humans and horses and has high lethality [30,31]. In Brazil, the isolation of animal cases was registered in the present decade [32]. EEEV was isolated from horses in the states of Bahia, Pernambuco, Pará, São Paulo, Minas Gerais, Rio de Janeiro, Mato Grosso and Paraná [33,34,35,36,37,38,39,40,41,42]. Currently, equine viral encephalomyelitis is sporadic in most Brazilian states [43].

Epidemiological studies carried out in the Brazilian Amazon were able to positively isolate EEEV [44]. Previously in humans, positivity was restricted to seroepidemiological surveys, with no neurological clinical manifestations associated with the virus. However, a fatal human infection was reported, and its occurrence was attributed to climate change and human encroachment on previously wild areas [45].

The EEEV transmission cycle occurs between birds and mosquitoes, and the genera *Aedes*, *Culex* and *Mansonia* have already been implicated in the transmission of EEEV among humans and horses, as they feed on birds and mammals [46]. Another form of transmission reported for this virus is by aerosol, which is of great concern due to the threat of its use as a possible bioterrorism agent [47].

#### 1.1.3. Western Equine Encephalitis Virus (WEEV)

Western equine encephalitis is a serious neurological disease capable of affecting humans and horses and is widespread in Canada, Latin America and the USA. The first isolation of this virus occurred in 1938 [48] in the USA. In Brazil, WEEV has been isolated in forests in Rio de Janeiro and Pará [49,50]. Known vectors belong to the genera *Aedes* and *Culex*, and viral isolation has also been obtained in chickens, pheasants, rodents, rabbits, ungulates, turtles, snakes and blood from wild birds [51].

The WEEV complex contains the following viruses: WEEV, AURAV, UNAV, TNTV, PIXV and MUCV. Some viruses originally isolated in the Amazon region have not yet been fully characterized [13] to justify their inclusion in a taxon. AURAV and UNAV were isolated only from mosquitoes, mainly *Aedes serratus* and *Psorophora ferox*, respectively. MUCV was isolated from all four types of sources (humans, wild animals, sentinels and arthropods). PIXV was isolated twice from mosquitoes (*Anopheles nimbus* and *Trichoprosopon digitatum*) and once from a wild rodent (*Proechimys guyannensis*). AURAV, found in South America, has not been linked to any disease in humans or animals.

TNTV was isolated from rodents (*Dasyprocta aguti* and *Orizomys* sp.) and mosquitoes (*Sabethes* spp.) in the Brazilian Amazon [46].

#### 1.1.4. Chikungunya Virus (CHIKV)

Chikungunya fever received this name because of the language spoken in East Africa, where the first patients were treated; the term means “those who bend over”, which corresponds to the position adopted by the patient due to the severe pain caused by CHIKV. There are also reports of sudden-onset fever greater than 38.5 °C and severe arthralgia or arthritis with acute onset, which may also include headache, muscle pain and skin rash [52].

The first record of the disease in Brazilian territory was described in 2010 in the state of Rio de Janeiro as a case imported from Indonesia. However, from 2014 onward, there were reports of autochthonous infections caused by CHIKV in Amapá and Bahia, with a consequent increase in the number of cases [53].

The disease is transmitted by *Ae. aegypti* and *Ae. albopictus* mosquitoes, which are widespread in all Brazilian states and are widely dispersed in urban areas facilitating the transmission and spread and increase of CHIKV in Brazil [54]. As a result, it is necessary to maintain care regarding the epidemiological surveillance of CHIKV and invest in studies related to the development of vaccines and educational campaigns to combat vectors.

#### 1.1.5. Pixuna Virus (PIXV), Mucambo Virus (MUCV), Una Virus (UNAV), Aura Virus (AURAV) and Triniti Virus (TNTV)

PIXV was isolated from arthropods in 1961 [36] in the state of Pará and from some human cases, one of which was related to a fatal laboratory infection [44], after which its isolation was not frequently reported; very little about this virus has been studied, with detection achieved only through serological records among horses in the state of Paraná [39] and among inhabitants of the Amazon region [55].

MUCV and many isolates from the Amazon are pathogenic to humans, and in this region, MUCV has been isolated from humans, sentinel animals, wild animals and mosquitoes. The UNAV, AURAV and TNTV were isolated from mosquitoes, with the latter also being isolated from a *Oryzomys* sp. rodent and maintaining the capacity to cause an unknown human infection until the present [13].

### 1.2. Flavivirus Genus

The *Flaviviridae* family consists of four genera (*Flavivirus*, *Pestivirus*, *Pegivirus* and *Hepacivirus*) comprising 89 viral species [19], of which more than 50 species are transmitted by arthropods, infecting mosquitoes or ticks [56]. Mammals and birds can also be their hosts, which can present hemorrhagic fever, neurological disease or even be asymptomatic. The *Yellow fever virus* (YFV), DENV, JEV, WNV and tick-borne encephalitis viruses are pathogenic to humans. Other members cause economically important diseases in domestic or wild animals. Other viruses in this group include SLEV, ROCV, CACV, ILHV, BUSV and IGUV [9,57].

The viral particle measures 40 to 60 nm in diameter and has a protein capsid with icosahedral symmetry, a single-stranded RNA genome, and positive polarity, approximately 11 kb in length [18]. Blood-sucking mosquitoes, such as *Ae. aegypti* and *Ae. albopictus* are implicated as transmitters of flaviviruses. These are able to mutate and/or adapt to new zoonotic cycles, re-emerging in places that have suffered environmental impacts as a result of human action in nature [58].

#### 1.2.1. Dengue Virus (DENV)

DENV is represented by four distinct serotypes: DENV-1 to DENV-4, which are maintained in cycles involving small primates and *Aedes* mosquitoes. In urban centers, this transmission occurs between mosquitoes and humans, with *Ae. aegypti* as the main vector, and *Ae. albopictus* and *Ae. polynesiensis* as secondary vectors. *Ae. aegypti* originated from the African continent and later spread to Asia and America by sea, arriving in Brazil in the mid-18th century by slave ships. The reports mentioned in the scientific literature mention the 1920s (20th century) as the beginning of epidemics that occurred in Brazilian territory, with the first reports occurring in the state of São Paulo in 1916 and Rio de Janeiro (Niterói) in 1923 [9].

In 1955, campaigns were carried out in an attempt to eradicate the *Ae. aegypti* vector in Brazil without success, causing viral circulation again in 1963 with the reemergence of DENV-2 and DENV-3 in several countries. At the end of the 1960s, the vector was already circulating in cities, such as Belém, Salvador and Rio de Janeiro [59].

In the 1980s, there was an expansion of dengue throughout Brazil, with the gradual emergence of the four serotypes, which resulted in the emergence of arbovirus epidemics that are still observed in the country [60]. Regarding the epidemiological profile of dengue in Brazil between 2014 and 2019, dengue cases were predominantly in the Southeast and Midwest regions, with a predominance of serotype 2 (DENV-2) [61].

Clinically, dengue manifests as an acute infectious disease characterized by fever, myalgia, headache, joint pain, retro-orbital pain, rash, thrombocytopenia, lymphadenopathy and leukopenia. Fever is sudden and high, persisting for two to seven days. Rash and pruritus are evident in the convalescent phase of the disease [62].

The most severe clinical phase is called dengue hemorrhagic fever (DHF), which is characterized by increased vascular permeability and results in hypovolemic shock, which can lead to tissue damage and multiple organ failure [62].

In addition to the bite of *Ae. aegypti* mosquitoes, there are also reports of vertical transmission and blood transfusion transmission. Studies, especially in endemic areas, such as the Amazon region, should be initiated in blood banks to determine the risk of infection for DENV and other flaviviruses for blood product recipients, especially in epidemic periods [63,64].

#### 1.2.2. Saint Louis Encephalitis Virus (SLEV)

SLEV is transmitted by mosquitoes of the *Culex* genus, and birds are considered amplifying hosts found in urban and rural environments. On the other hand, men and domestic animals are hosts that show symptoms but do not develop significant viremia [65].

Reservoirs of this virus mainly include birds, primates, marsupials and other wild animals. The first record of the isolation of this virus was in the USA from an insectivorous bat, and in Brazil [66] isolation was carried out in 1960 from *Sabethes belisarioi* mosquitoes collected on the Belém–Brasília highway [67].

In Brazil, only three cases of SLEV in humans were reported in the 1970s, two in the northern region and a third identified in the municipality of São Pedro/SP in 2004 [67]. More recently, the detection of SLEV was described in humans in the states of São Paulo and Mato Grosso during outbreaks of DENV and ZIKV [68].

Most reported cases of SLEV go unnoticed because they are asymptomatic, subclinical or have symptoms similar to those of a common cold. The main signs and symptoms observed include fever, headache, myalgia, nausea/vomiting and drowsiness [69].

The illness caused by SLEV can range from mild symptoms, including fever and headache, to severe illness, such as meningitis and encephalitis [9]. The percentage of case report fatalities can range from 5% to 20%; however, the numbers are even higher in the elderly population [70].

#### 1.2.3. West Nile Virus (WNV)

WNV was identified in the West Nile district of Uganda [71]. Over the years, it has also been observed in Africa, Europe, North America, the Middle East and Asia [72,73]. In Brazil in 2009, antibodies against WNV were detected in horses in the state of Mato Grosso; in subsequent years, serological evidence continued to be detected in poultry, horses and humans [74,75,76]. Subsequently, another study carried out in the same region detected the presence of neutralizing antibodies not only against WNV but also against other flaviviruses in horses, sheep and alligators, showing wide circulation of these viruses in the region.

However, it was only in 2018 that the virus was identified and isolated from the central nervous system (CNS) of horses with neurological disease in the state of Espírito Santo [77,78]. Additionally, the identification of positive cases among humans and horses in the states of Piauí, São Paulo and Minas Gerais indicated the need to expand the study and epidemiological surveillance of WNV in the country [79,80,81]. Equine surveillance by veterinarians is essential to provide constant information about the circulation of WNV in the national territory [81].

Symptoms vary in each host, ranging from the asymptomatic form, common in horses and humans, to neurological manifestations in birds, such as disorientation, ataxia, tremors and head tilt, followed by death. Approximately 20% of humans who develop symptoms have nonspecific clinical signs, such as fever, body pain and myalgia, and a smaller number of those affected may have the neuroinvasive form, characterized by meningitis, encephalitis and paralysis progressing to death [82,83].

WNV transmission is maintained in an enzootic cycle, that is, between arthropods and wild birds, but it can also infect horses and mammals, including humans, the latter being considered accidental and final hosts because they develop a low viral load, which does not allow them to be involved in the transmission cycle. Other forms of transmission can be transovarian, which favors the permanence of the virus in mosquitoes, provided that there are favorable conditions for its maintenance and consequent transfer to progeny [82,84], organ transplantation, placental route, and blood transfusion [85,86,87,88].

In 2002, the first case of suspected WNV infection after an obstetric procedure in a patient who received blood and blood products was reported [89]. The worldwide prevalence of arboviruses has grown dramatically in recent decades, and in areas where these diseases are endemic, transmission by transfusion has rarely been investigated. Concerns about the transmission of arboviruses by blood transfusion increased after the documentation of the transmission of WNV by transfusion in the USA. In Brazil, studies indicated that asymptomatic individuals can be blood donors and serve as a source of virus dissemination in the community [63].

#### 1.2.4. Cacipacoré Virus (CACV), Ilhéus Virus (ILHV) and Bussuquara Virus (BUSV)

CACV was first isolated from birds (*Formicarius analis*) in 1977 in an Amazonian area in the state of Pará. The presence of antibodies against this arbovirus was detected in free-living monkeys captured in the state of Mato Grosso do Sul, with two animals showing antibodies against flaviviruses and one showing a monotypic reaction against CACV. More recent studies reported the isolation of CACV from mosquitoes collected in the Amazon region and from human blood in the state of Rondônia [90,91].

ILHV was isolated from a pool of mosquitoes of the genera *Aedes* and *Psorophora* during an epidemiological investigation of yellow fever in the city of Ilhéus, state of Bahia, in 1947 [92]. Birds are the main vertebrate hosts, and humans are considered accidental and terminal hosts, as they develop low viremia. ILHV was detected in invertebrates (*Aedes* spp. mosquitoes) and vertebrates in the Pantanal region and in the state of Pará, São Paulo, respectively [9,93,94].

BUSV was originally isolated from primate blood near the city of Belém in 1959 [95]. The transmission cycle occurs between mosquitoes of the *Culex* genus and rodents, although birds can act as reservoirs. In 1971, the first case of a human febrile illness by BUSV was reported in Panamá [96]. In 2015, sera from nonhuman primates from Goiânia in the Brazilian Midwest were positive for BUSV [97].

The IEC was created in 1936, with the name of North Institute for Experimental Pathology Institute, by the state government of Pará, and in 1942, it was federalized, becoming a scientific research body of the Special Service Foundation for Public Health. In 1954, with the support of the Rockefeller Foundation, studies on arboviruses in the Amazon region were hosted at the IEC, which brought innovations in field and laboratory procedures that have contributed in a decisive and invaluable way to public health and the scientific community during these five decades.

The objective of this work is to present the encephalitogenic viruses that have been registered in the Department of Arbovirology and Hemorrhagic Fevers (SAARB) of the IEC over the past six decades.

## 2. Methods

For viral isolation, samples such as blood, serum, suspensions of human or animal viscera and arthropods were used. The aforementioned samples (viscera and arthropods) were ground in 1000 μL of diluent buffer (5% fetal bovine serum (FBS)), penicillin (100 U/mL), streptomycin (100 μg/mL) and gentamicin (0.05 mg/mL) in TissueLyser equipment (Qiagen, Hilden, Germany) using 5-mm diameter “beads” (stainless steel microspheres) at 26,000 movements per minute for 4 min, followed by freezing at −70 °C and later centrifugation at 10,000 rpm for 10 min at 4 °C. Then, each sample was centrifuged at 15,652× *g* for 10 min at 4 °C. After centrifugation, the samples remained at that temperature until inoculation into a newborn Swiss mouse at 2 to 3 days of life. The animals were inoculated intracerebrally with a volume of 0.02 mL of the suspensions with the aid of a 1 mL syringe and a 25 × 6 disposable needle. This inoculation step was also valid for the other biological samples. The cages that contained the animals that were to be used were identified with batch numbers and internal records of the sample, material type and inoculation date. They contained a lactating female and 6 newborn Swiss albino mice (two days old) [98].

After inoculation, the animals were observed daily for up to 21 days, and records were made on a card (inoculation card).

Mice that showed signs of disease were collected and stored in a freezer at −70 °C until laboratory tests were carried out, while those that did not show signs were disposed of following institutional protocols.

The Laboratory of Viral Isolation/SAARB/IEC has its own Vero (renal cells of *Chlorocebus aethiops*) and C6/36 (*Ae. albopictus*) cells which were constantly used during these decades.

Vero cells were maintained in Medium 199 supplemented with glutamine (2 mM), sodium bicarbonate (3 mM), penicillin/streptomycin (104 IU/mL), fungizone (2.5 mg/mL) and 10% FBS for cell growth, with the amount of FBS being reduced to 5% for cell maintenance. The cells were trypsinized every 7 days, and the medium was replaced every 2 days and kept at 37 °C under a humid atmosphere of 95% H2O and 5% CO2. For the C6/36 cells, L-15 medium was used, trypsinization was performed manually, and the cells remained in a room with a temperature of approximately 25 °C [99].

Briefly, 100 µL of each diluted viral sample was inoculated into monolayers of the aforementioned cells propagated in 16 × 125-mm tubes and then incubated at 37 °C and 28 °C for a period of 1 hour (adsorption), and then 1.5 mL of overlay medium (maintenance medium 199 and L-15) was added to the cells. Cell cultures were observed daily to monitor possible cytopathogenic effects (CPEs) with the aid of an Axiovert S100 microscope (Zeiss, Oberkochen, Germany). Negative controls were made from the inoculation of uninfected cell fluids. The observation of infected cultures was performed with the aid of an Axiovert S100 microscope (Zeiss).

## 3. Result and Discussion

**Figure 2:**
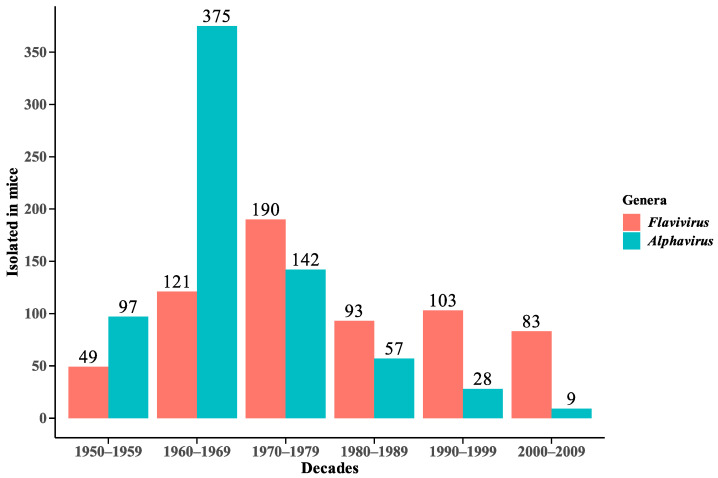
Distribution of *Flavivirus* and *Alphavirus* viral isolates in mice in decades from 1954 to 2009.

The samples isolated in mice registered by the SAARB/IEC from 1954 to 2009 correspond to 639 flaviviruses and 708 alphaviruses (Figure 2).

**Figure 3:**
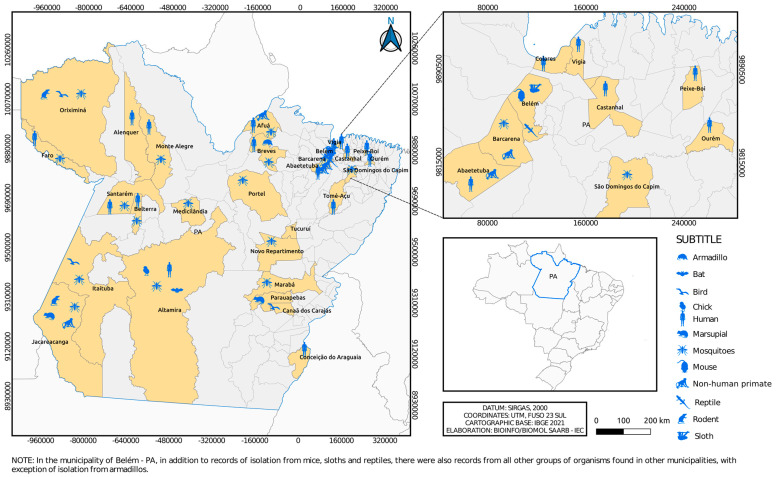
Municipalities in Pará state where flaviviruses were isolated over 6 decades by the SAARB/IEC.

**Figure 4:**
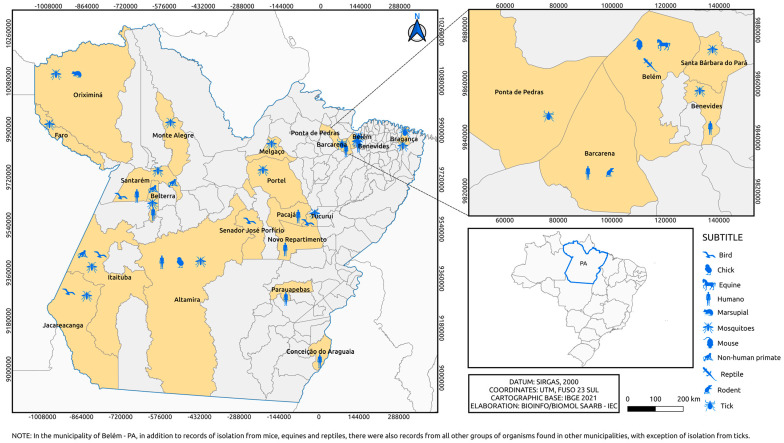
Municipalities in Pará state where alphaviruses were isolated over 6 decades by the SAARB/IEC.

**Figure 5:**
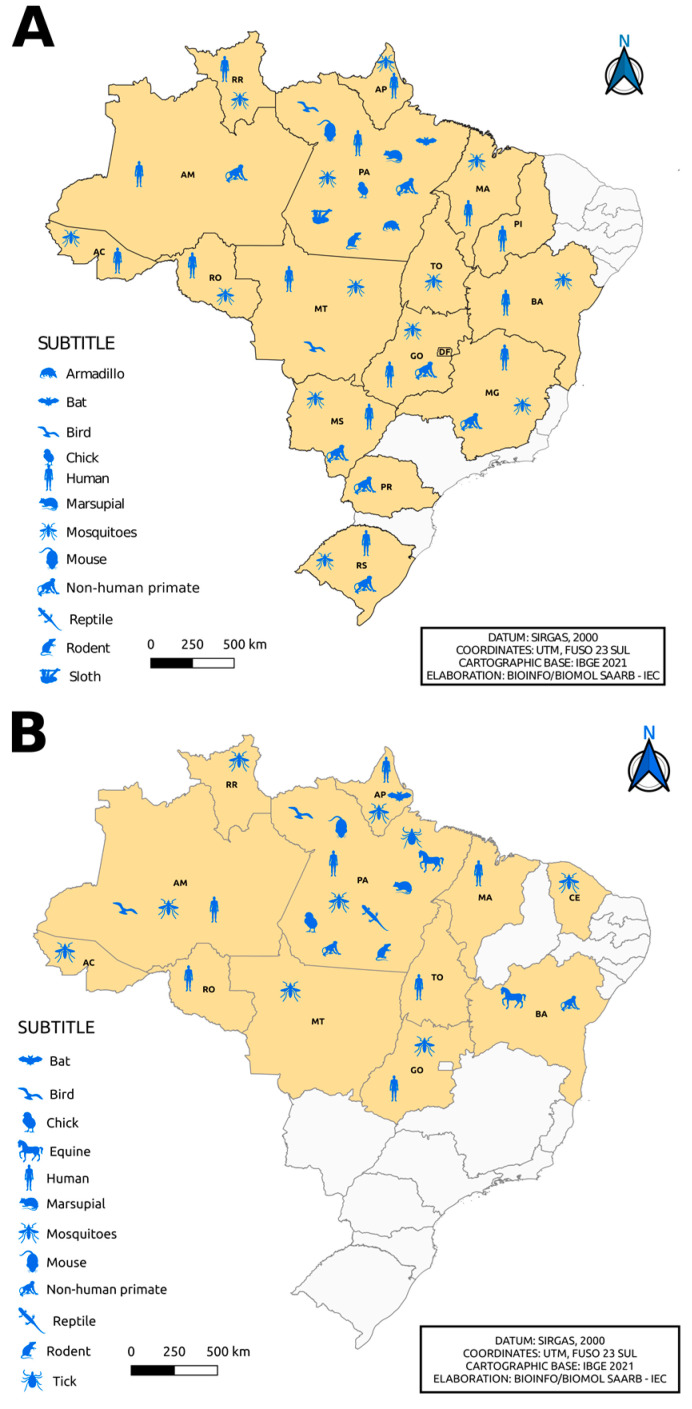
Distribution of the incidence of *Flavivirus* (**A**) and *Alphavirus* (**B**) reservoirs by federative unit of Brazil.

The isolates are geographically distributed among locations in the State of Pará (Figure 3 and Figure 4), with records in other states of the Brazilian federation (Figure 5). In the State of Pará, the records include forest areas in the region, such as Mata de Utinga and Uriboca, around highways, such as Belém–Brasília, Transamazônica and Santarém–Cuiabá, in addition to areas where research and surveillance projects are developed by the IEC, such as the Jari project and the locations of the Ecological Research Area of Guamá (APEG), Bosque Rodrigues Alves, Airport Road, the colony of Guamá, farms (Matinha, Oriboca-Pirelli, Piramucal), the Agronomic Institute of the North (IAN) and the Institute of Agricultural Research and Experimentation (IPEAN). Additionally, in the state itself, arbovirus records of these two genera comprise localities in inland cities, such as Abaetetuba, Afuá, Água Preta, Alenquer, Altamira, Barcarena, Belém, Benevides, Bragança, Breves, Cachoeira Porteira (Cacipacoré, Oriximiná), Castanhal (Alto do Capim), Catu, Faro, Itaituba, Marabá, Marajó (Ponta de Pedras), Medicilândia, Monte Alegre, Mucambo, Tucuruí, Ourém, Pacajá, Parauapebas, Portel, Santa Bárbara, Bragança and Santarém.

The isolation of flavivirus and alphavirus occurred in other locations in Brazilian states, such as Serra do Navio—ICOMI (Amapá State), Aripuanã (Mato Grosso State), Boca do Acre (Acre State), Uruaçu (Goias State), Tocantins State, Macapá (State of Amapá), Bom Jardim (State of Maranão), Instituto Baiano de Biologia (IBIO/UFBA) (State of Bahia), Manaus (State of Amazonas) and states of Rio Grande do Sul, Mato Grosso, Mato Grosso do Sul, Paraná and Minas Gerais, in addition to a registration abroad in the city of Iquitos (Peru). In the northern region of the country, specifically in the state of Pará, the isolation of flaviviruses in armadillos and sloths (Figure 5A) and alphaviruses in ticks and horses (Figure 5B) was observed, differing from other Brazilian states. In addition, viral isolates of alphavirus were noted in horses in the state of Bahia, with a notable absence of isolation of viruses of this genus, considering the period evaluated, in the states of Minas Gerais, Mato Grosso, Paraná, Rio Grande do Sul and Piauí (Figure 5B).

**Figure 6:**
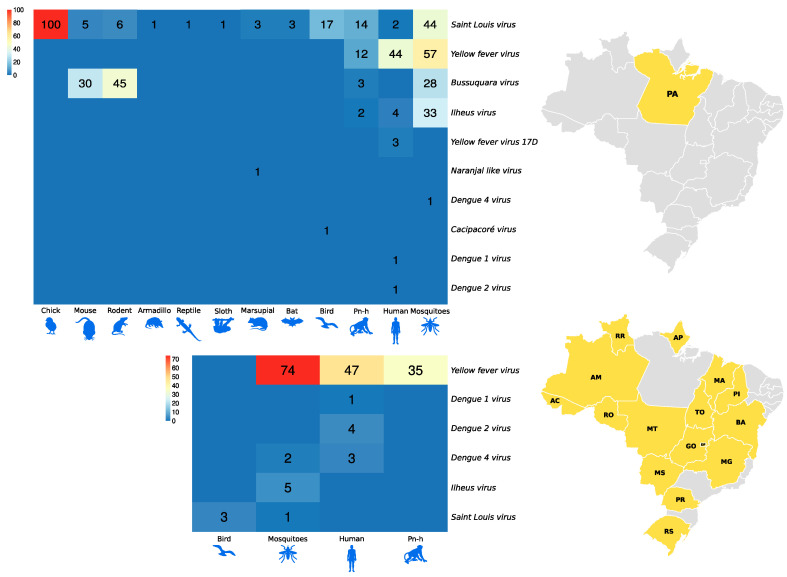
Distribution of flavivirus isolates in mice according to the animal model of origin. The larger heatmap is associated with isolates in the state of Pará. The smaller heatmap is associated with isolates from other Brazilian states.

**Figure 7:**
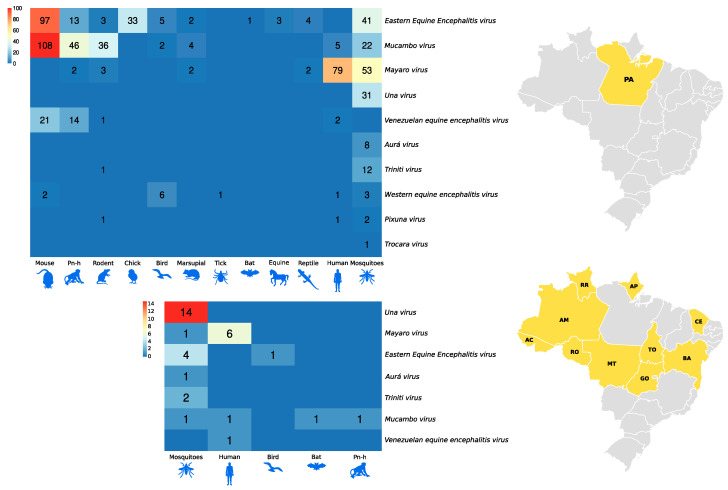
Distribution of alphavirus isolates in mice according to the animal model of origin. The larger heatmap is associated with isolates in the state of Pará. The smaller heatmap is associated with isolates from other Brazilian states.

Figure 6 and Figure 7, as well as Figure 1, show the amounts of arboviruses with encephalitogenic potential isolated in mice and distributed according to the animal model of origin, with records for the State of Pará and for other Brazilian states, accounting for: flaviviruses [13 DENV, 202 SLEV, 106 BUSV, 44 ILHV, and 1 CACV] and alphaviruses [39 VEEV, 207 EEEV, 13 WEEV, 148 MAYV, 4 PIXV and 227 MUCV]. In addition, a total of seven alphaviruses and four flaviviruses were partially identified. In both viral families studied, isolation was achieved in animals, arthropods and humans, with declining success. Approximately 293 viruses are in the families *Togaviridae* (9), *Rhabdoviridae* (3), *Sedoreoviridae* (4), *Picornaviridae* (21), *Flaviviridae* (4), *Peribuniviridae* (225) and *Arenaviridae* (27) with incomplete characterization; 8 are partially identified and therefore have unknown pathogenesis: *Iriri virus*, *Uruará virus*, *Galibi virus*, *Codajás virus*, *Cajazeira virus*, *Marajó virus*, *Cantá virus*, *Tracambé virus* and *Naranjal virus*.

**Figure 8:**
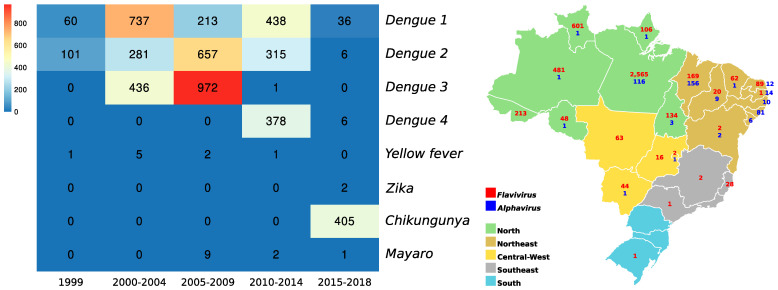
Distribution of alphavirus and flavivirus isolates between 1999 and 2018, from human samples and isolated exclusively using the cell culture technique. The heatmap illustrates the distribution of isolated viruses as a function of years in 5-year intervals, and the map shows the numbers of isolates in this period and distributed throughout the 5 regions of Brazil.

Between 1999 and 2018, it was possible to isolate from human samples and exclusively using the cell culture technique, 5044 arboviruses (of a total of 5065) with neuroinvasive potential, distributed in 1484 DENV-1, 1360 DENV-2, 1409 DENV-3, 384 DENV-4, 02 ZIKV and 405 CHIKV. In this period, the largest number of *Flavivirus* isolates was from the North region of the country, while the largest number of *Alphavirus* isolates came from states in the Northeast region, in addition to the absence of confirmed isolates for this genus in the Southeast and South regions (Figure 8).

The technique of viral isolation from mice and using mosquitoes as an animal model of origin was used by the IEC from 1966 to 2012, and with it, it was possible to isolate about 647 viruses among known arboviruses with encephalitogenic potential and even new ones for science. Isolation records using mosquitoes as an animal model of origin restarted again from 2018, exclusively using C6/36 cell culture (Ae. albopictus), and in the period between 2018 and 2022, 16 isolates were positive for *Flavivirus* and 13 isolates for *Alphavirus*, totaling in the period 1966 to 2022, using both techniques, 676 isolations from species of genera, such as *Aedes*, *Anopheles*, *Culex*, *Mansonia*, *Psorophora* and *Sabethes*, with records for AURAV, BUSV, DENV, EEEV, ILHV, MAYV, MUCV, PIXV, SLEV, TNTV, UNAV and WEEV.

## 4. Conclusions

The growing deforestation in the 20th century was associated with the challenge of eradicating arbovirus-transmitting vectors and the uncertain diagnosis of these viruses, whether due to antigenic similarity, causing cross-reactions (in arbovirus-endemic areas) in laboratory tests or similarity of symptoms among patients, constituting old and recurring problems in public health [10,100]. Concomitantly, disorderly urbanization, with the accumulation of garbage in urban areas, favors the proliferation of arbovirus vectors and contributes to the emergence and/or re-emergence of diseases in Brazilian territory, especially arboviruses caused by flaviviruses and alphaviruses [101].

Many of the arboviruses isolated by the IEC in epidemiological research on the circulation of arboviruses, especially in the Amazon region, led to a vast biological collection, with approximately 10,000 arboviruses, including those unknown to science, many of which still have incomplete taxonomic characterization [102].

Over all these years, the information generated by the SAARB/IEC has revealed ecological, environmental and epidemiological information about arboviruses. Studies of this nature are relevant, particularly in the Amazon region, as it has favorable conditions for the maintenance cycle of these agents, demonstrating an ideal environment for the emergence or re-emergence of arboviruses [98].

Clinical manifestations may be absent at the time of infection, which causes unrecognized viral spread through the free movement of individuals, even to nonendemic regions, a situation that imposes the need for constant surveillance of arboviruses [103,104]. This incessant work in public health helps promote the detection and knowledge of the circulation of viruses, thus enabling the identification of the potential agents responsible for outbreaks and epidemics. The prevention of arboviruses and vector control are the most effective measures in the current scenario where there is no vaccine or specific treatment available for most arboviruses [4].

In light of the epidemiological scenario demonstrating the simultaneous circulation of arboviruses with encephalitogenic potential that are of public health importance, in 2017, the Ministry of Health changed the Sentinel Surveillance Manual for Arbovirus Neuroinvasive Diseases to expand the surveillance of neuroinvasive diseases caused by arboviruses [4].

In light of what was observed in this study, it is possible to conclude that the isolation of ZIKV coincided with the first reports published by the Pan American Health Organization (PAHO), examining physical and emotional effects in the affected population, especially among women of childbearing age due to congenital infections that result in fetal malformations and deaths [105,106].

In light of the above, the need for constant epidemiological surveillance has become vital to investigate the circulation of arboviruses, many of which are still unknown. This is particularly true for the Amazon region which is a true hotspot for known viruses and for those completely new to science.

With over 80 years of experience, the IEC has collaborated with the Ministry of Health in reporting outbreaks and epidemics, whether by reporting cases, implementing training programs or developing human resources that will contribute to the reduction in arbovirus cases [104].

We reiterate the concern regarding the need for urgent actions to combat forest degradation that influences the spread of diseases transmitted by arthropods and their adaptation to the domestic environment with the possibility of emerging or re-emerging zoonoses [107].

Regarding the data presented in this review, it is of great importance to understand that health education constitutes a mechanism through which the population is encouraged to modify behaviors associated with prevention.

Currently for most arboviruses, no vaccine is available, and the cycle of sylvatic transmission of viruses cannot be prevented. Thus, preventive measures are necessary. These include avoiding contact in the areas of occurrence, particularly rural and wild forested areas and riverbanks, and minimizing exposure to vector bites, either by means of individual protection (use of repellents and full-length clothes) or collective protection (use of curtains and mosquito nets) [4].

There are numerous arboviruses present in different environments worldwide, especially in the Amazon, due to the favorable conditions found there; more virulent forms of these viruses are constantly emerging and invading new habitats. Therefore, knowing the epidemiology of these arboviruses is of fundamental importance to predict future mechanisms of emergence and/or re-emergence, thereby implementing measures to control and prevent the spread of arboviruses in urban areas. In addition to encephalitogenic viruses, there are many arboviruses with little information available in the literature [3,4,5]. It is only a matter of time before we see the emergence of more virulent phenotypes that can spread to a susceptible population that has no immunological memory for these viruses. The ZIKV pandemic is a recent classic example that showed us the power of arbovirus dispersion and, most importantly, its association with congenital malformations associated with ZIKV infection [105,106]. Unfortunately, the prevention of infection from the majority of arboviruses depends almost exclusively on efforts to control the populations of potential vectors, whether through the use of repellents, protective clothing or by raising awareness to reduce outbreaks at breeding sites. Despite great efforts to develop vaccines against arboviral infections, especially those that cause severe encephalitis, only a few have been successful [108].

The search for vaccines is a priority, and several live attenuated vaccines based on modern molecular biology techniques are in full pharmaceutical development. Some, such as the ChimeriVax candidates developed against WNV, Japanese encephalitis and dengue, have already shown good results in terms of safety and immunogenicity [108]. Our collection shows the great viral diversity that has already been isolated in our country, which can contribute to the development of vaccines and promising antiviral drugs aimed at isolated and known viruses.
